# Association between biomarkers of tissue inflammation and progression of osteoarthritis: evidence from the Rotterdam study cohort

**DOI:** 10.1186/s13075-016-0976-3

**Published:** 2016-04-01

**Authors:** Fatemeh Saberi Hosnijeh, Anne Sofie Siebuhr, Andre G. Uitterlinden, Edwin H. G. Oei, Albert Hofman, Morten A. Karsdal, Sita M. Bierma-Zeinstra, Anne C. Bay-Jensen, Joyce B. J. van Meurs

**Affiliations:** Department of Internal Medicine, Erasmus University Medical Center, P.O. Box 2040, Rotterdam, 3000 CA Netherlands; Biomarkers and Research, Nordic Bioscience, Herlev, Denmark; Department of Epidemiology, Erasmus University Medical Center, Rotterdam, The Netherlands; Department of Radiology, Erasmus University Medical Center, Rotterdam, The Netherlands; Department of General Practice, Erasmus University Medical Center, Rotterdam, Netherlands; Department of Orthopedics, Erasmus University Medical Center, Rotterdam, Netherlands

**Keywords:** Osteoarthritis, Inflammation, Biomarker, CRP, Prospective cohort

## Abstract

**Background:**

We aimed to investigate the prognostic value of two biomarkers of tissue inflammation, matrix metalloproteinase-dependent degradation of C-reactive protein (CRPM) and connective tissue type I collagen turnover (C1M), on the incidence and progression of radiographic osteoarthritis (OA) in the Rotterdam Study, a prospective cohort. Moreover, the independent effect of these biomarkers with respect to the established biomarkers of OA progression, like urinary type II collagen degradation (uCTX-II) and serum cartilage oligomeric protein (COMP), was evaluated.

**Methods:**

Serum levels of C1M, CRPM, COMP and CRP of 1335 participants aged >55 years were measured in fasting serum using ELISA. The commercial ELISA detecting CTX-II was used in urine. Radiographs at baseline and 5-year follow-up were scored for OA stage by Kellgren-Lawrence grade. The associations between progression and incidence of OA and the baseline biomarkers were examined using logistic regression and generalized estimating equations adjusted for age, sex, BMI, and possible other confounders.

**Results:**

The uCTX-II, COMP, and CRP concentrations were associated with the incidence and progression of OA. Moreover, OA progression was positively associated with CRPM (OR = 1.3, *p* = 0.01) and CRP (OR = 1.3, *p* = 0.01) levels with similar effect size as uCTX-II (OR = 1.3, *p* = 0.01) and COMP (OR = 1.2, *p* = 0.02). CRPM had prognostic value for progression of OA independent from the uCTX-II and COMP.

**Conclusions:**

Our study confirmed the associations between uCTX-II and COMP concentrations and OA progression. Importantly, we showed for the first time that CRPM predicts the risk of OA progression independent of the established biomarkers uCTX-II and COMP.

**Electronic supplementary material:**

The online version of this article (doi:10.1186/s13075-016-0976-3) contains supplementary material, which is available to authorized users.

## Background

Osteoarthritis (OA), the most common form of arthropathy, is characterized by alteration of joint structure including progressive cartilage destruction, synovial inflammation, and changes to the subchondral bone [1]. The etiology of OA is not well understood although the knowledge in this respect has accumulated during the past decades. Beside genetic variation and biomechanical mechanisms [1], altered lipid metabolism [2] and inflammation [3] might also be important drivers of the molecular mechanism underlying OA.

It is clear that OA is heterogeneous in its etiology and disease course. Recent efforts are now focused on identifying subgroups of patients with distinct disease pathology, which will allow the development of new targeted therapies [1]. Circulating biochemical markers (biomarkers) have the potential to serve as a measure of the different pathological processes linked to OA. However, very few biomarkers have been identified that can predict the course of OA. Up to now, only urinary C-terminal telopeptide of collagen type II (uCTX-II) and serum cartilage oligomeric protein (COMP) (both markers of cartilage and bone metabolism) have shown discriminative ability for diagnosis and prognosis of OA [4–10].

Compared to rheumatoid arthritis (RA) or seronegative spondyloarthritis, inflammation is less prominent in OA. There is no marked infiltration of inflammatory cells into joint tissues, and the synovial fluid usually contains few neutrophils [11]. C-reactive protein (CRP), a systemic biomarker for inflammation, has been shown to be elevated in some OA patients yet the evidence is conflicting [12]. Recent studies have suggested that local inflammation plays a prominent role in the pathogenesis, symptoms, and progression of OA [3, 13, 14]. Recently, a newly developed CRP measure was described. Matrix metalloproteinase (MMP)-dependent degradation of CRP (CRPM) can be measured in serum to quantify CRP fragments released from the inflamed tissue, after CRP has been synthesized in the liver and deposited in the joint and degraded by the proteolytic burden [15]. It has been shown that MMP-degraded CRP provides a more discriminative diagnostic potential compared to that of full-length CRP in ankylosing spondylitis (AS) patients [16].

Synovial inflammation (synovitis) is a common characteristic of inflammatory OA and is believed to stimulate the connective tissue turnover. Type I collagen, the most abundant structural collagen of the human body, is a collagen of the connective tissue, including the synovial membrane. The collagen biomarker C1M is a measure of MMP-driven soft tissue destruction [17]. In a recent study among RA patients, C1M levels were associated with progression of RA and were also shown to correlate with RA activity [18]. Moreover, elevated levels of C1M were found in OA patients with higher CRP [15] and CRPM levels [16]. It seems that inflammation is important in disease pathology in a subset of the OA population; however, the pathology of this subset is not well described and few longitudinal analyses of these patients have been presented [19, 20].

Due to a general lack of consistent evidence, differences between the populations studied (clinical trials vs. population-based cohorts), and differences in sample collection, future research is needed to validate the existing OA markers and to identify new candidates. The aims of the present study were to explore the prognostic value of two biomarkers of tissue-inflammation: C1M and CPRM. In addition, we examined the extent to which these two biomarkers could be considered to be independent of well-known markers of uCTX-II and COMP and demographic characteristics such as age, sex, and body mass index (BMI) for incidence and progression of radiographic OA in a large population of men and women. Moreover, we investigated whether CRPM has prognostic value for OA progression compared to the full-length CRP.

## Methods

### Study population

The Rotterdam Study (RS) is a population-based prospective cohort study ongoing since 1990 to investigate the occurrence and determinants of diseases in an aging population [21]. The Rotterdam Study consists of three subpopulations. The Rotterdam Study-I (RS-I) is the first cohort of 7983 persons aged 55 years and older living in the Ommoord district of Rotterdam in the Netherlands [21]. The RS-II started in 2000 when 3011 participants were recruited into the study when they became 55 years of age or moved into the study district. In 2006, a further extension of the cohort was initiated, the RS-III, in which 3932 subjects, aged 45–54 years, were included. The study has been approved by the institutional review board (Medical Ethics Committee) of the Erasmus Medical Center and by the review board of The Netherlands Ministry of Health, Welfare and Sports and all participants gave written informed consent.

Baseline measurements were obtained through a home interview and visits to the research center for physical examinations and laboratory assessments. Blood samples were drawn by venous puncture and stored at –20 °C at baseline. Weight-bearing anteroposterior radiographs of the knee and hip were obtained at baseline and after 5 years of follow-up. Radiographs were acquired with the knee extended and the patella in a central position. Radiographs of the pelvis were obtained with both feet in 10° internal rotation and the X-ray beam centered on the umbilicus. The present study includes RS-II cohort’s participants for whom knee and hip radiographs at baseline and 5-year follow-up were available and scored. Subjects without baseline (n = 114) and follow-up radiographs (n = 1358), without informed consent (n = 1), without all biomarker data (n = 187), and subjects with AS (n = 2), with RA (n = 11), and with total joint replacement (TJR) due to fracture (n = 3) were excluded from the study. Therefore, out of 3011 participants, 1335 subjects were included in the current study.

### Outcome assessment

Radiographs were scored for the presence of a TJR and OA of the hip and knee according to the Kellgren and Lawrence (KL) score. Radiographic OA (which we refer to here as OA) was defined as a KL score ≥2 of one or both joints or a TJR [22–25]. In addition, we defined TJR as grade 5. The incidence of knee and/or hip OA was defined as a combination of KL <2 at baseline and KL ≥2 at follow-up. As there is no consensus on the definition of progression, we combined incidence and progression in one definition for the overall progression of osteoarthritis. This was defined as an increase in the KL score between baseline and follow-up of ≥1. In the case of a baseline score of zero, overall progression was defined as an increase of ≥2. Patients with scores of 4 and 5 at baseline were left out of the progression analysis. Controls were free of OA at the joint site studied but were allowed to have OA at other joint sites. For example, if knee OA was studied, controls had to be free of knee OA but were allowed to have hip OA. In total OA analyses, controls were free of both hip and knee OA.

Joint pain was determined to be present based on the answer (yes/no) to the questions if participants had had persistent joint pain and stiffness in the last 6 weeks.

### Quantification of biomarkers

In order to ensure the reproducibility and performance of the assays, three genuine urine or serum samples, in addition to the kit controls, were added as quality controls on each microtiter plate, and the entire plate was rerun if any of the genuine controls were determined to have a concentration >20 % of the predetermined value.

#### uCTX-II measurement

Subsequent to overnight fasting, urine samples were obtained from all subjects at baseline and kept frozen at –20 °C. Monoclonal antibody mAbF46, specific for CTX-II fragments, was used in a competitive enzyme-linked immunosorbent assay (ELISA) (Immunodiagnostic Systems Nordic, Copenhagen S, Denmark) following the instructions of the manufacturer. The concentration of uCTX-II (in ng/l) was standardized to the total urine creatinine (mmol/l), and the units for the corrected uCTX-II concentration are ng/mmol [7].

#### COMP measurement

Serum COMP (COMP®, AnaMar, Göteborg, Sweden) were measured using enzyme-linked immunosorbent assays based on a monoclonal antibody.

#### CRPM, C1M measurements

Biomarkers were analyzed from fasting serum by Nordic Bioscience, Herlev, Denmark. The markers were measured by validated ELISAs applying neoepitope-specific monoclonal antibodies (Nordic Bioscience, Herlev, Denmark). The technical data (reproducibility and stability) for the assays are described in the published technical articles on the assays. The technical data are available in the following articles: C1M [17], and CRPM [16].

#### CRP measurement

High-sensitivity (hs)-CRP was measured using Rate Near Infrared Particle Immunoassay (Immage Immunochemistry System; Beckman Coulter, Brea, CA, USA). This method can accurately measure protein concentrations from 0.2–1440 mg/l with a within-run precision <5.0 %, a total precision <7.5 %, and a reliability coefficient of 0.995 [26].

### Statistical analyses

Missing values of biomarkers [C1M: n = 39 (2.9 %); CRPM: n = 38 (2.8 %); CTX-II: n = 116 (8.7 %); COMP: n = 12 (0.9 %); CRP: n = 35 (2.6 %)], BMI (n = 6, 0.4 %), alcohol intake (n = 6, 0.4 %), smoking status (n = 1, 0.08 %), education (n = 20, 1.5 %), and diabetes (n = 5, 0.4 %) were imputed based on a maximum likelihood estimation method accounting for the correlation structure within the data [27]. In all analyses, levels of markers were log_10_-transformed to normalize their distributions. Standardized scores (Z score) were made for continuous variables; therefore the odds ratio (OR) is expressed as percentage of change per one standard deviation.

Pearson correlation coefficients were calculated to evaluate the correlation between biomarkers. A logistic regression model adjusted for confounding variables was used to calculate OR and 95 % confidence interval (CI) for incidence and overall progression of OA in relation to each biomarker. Age, gender, BMI [weight (kg)/height (m^2^)], and presence of radiographic OA were included as confounding variables. The effect of diabetes, current smoking (self-reported), educational level, and alcohol intake (current, former, never) as potential confounding variables were examined, but did not appreciably change the risk estimates (less than 10 % change in the estimates) and, therefore, were not included in the final models. Moreover, through simultaneous modeling, we investigated whether the biomarker findings are independent of one another. Generalized estimating equation (GEE) models were further used for a joint-based analysis of knees and hips to fit the models for correlations between the right and left extremity in each individual. Evidence of statistical interaction of biomarkers with sex and age was evaluated by including cross-product interaction terms in the corresponding multivariable models. We further evaluated the association between the biomarkers and baseline joint pain among individuals with OA at baseline. In order to assess the discriminating power of the biochemical markers studied we generated receiver operating characteristic (ROC) curves for each model. Area under the curve (AUC) of the models including age, sex, and BMI after adding joint pain, baseline KL score, and the biomarkers were evaluated. Statistical analyses were performed using SPSS (IBM SPSS Statistics 21; IBM Corp., Armonk, NY, USA).

## Results

The descriptive characteristics of study participants are presented in Table 1. Our study population was slightly younger, less obese, less diabetic, and drank more alcohol than total population. OA cases at baseline and follow-up were older, more obese and diabetic, and smoked less than subjects who remained healthy at follow-up time.

**Table 1:** General characteristics of the study participants

Baseline variables	Total cohort, n = 3011	Study subjects, n = 1335			
		All subjects at baseline	OA at baseline, n = 238	OA at follow-up, n = 326	No OA at follow-up, n = 955
Female, n (%)	1694 (56)	743 (55.7)	144 (60.5)	195 (59.8)	519 (54.4)
Age^*^	65.2 (8.43)	63.1 (6.48)	66.4 (7.90)	66.02 (7.72)	62.13 (5.71)
Body mass index, kg/m^2*^	27.3 (4.23)	27.04 (3.85)	27.96 (4.11)	28.01 (4.29)	26.69 (3.65)
Current alcohol drinker, n (%)	2455 (81.5)	1146 (85.8)	205 (86.1)	277 (85)	822 (86.2)
Current smoking, n (%)	692 (23)	298 (22.3)	37 (15.5)	59 (18.1)	226 (23.7)
Low level of education, n (%)	424 (31.8)	999 (33.9)	74 (31.1)	103 (31.6)	298 (31.2)
Diabetes, n (%)	182 (6.0)	68 (5.1)	12 (5.0)	21 (6.4)	42 (4.4)
Knee pain, n (%)	725 (24.1)	328 (24.6)	96 (40.3)	128 (39.3)	185 (19.4)
Hip pain, n (%)	458 (15.2)	207 (15.5)	48 (20.2)	62 (19)	132 (13.8)
Knee OA, n (%)	339 (11.3)	185 (13.9)	185 (78.1)	185 (56.7)	-
Hip OA, n (%)	141 (4.7)	68 (5.1)	68 (28.7)	68 (20.9)	-
uCTX-II^*^, ng/mmol	-	2.3 (0.23)	2.42 (0.27)	2.40 (0.26)	2.27 (0.22)
COMP^*^, U/L	-	1.03 (0.10)	1.06 (0.10)	1.06 (0.10)	1.02 (0.10)
CRP^*^, mg/l	-	0.02 (0.49)	0.07 (0.48)	0.12 (0.49)	-0.02 (0.48)
CRPM^*^, ng/ml	-	1.01 (0.18)	1.01 (0.18)	1.02 (0.18)	1.01 (0.17)
C1M^*^, ng/ml	-	1.59 (0.17)	1.60 (0.18)	1.61 (0.18)	1.58 (0.17)

*OA* osteoarthritis, *uCTX-II* (urinary) type II collagen degradation, *COMP* cartilage oligomeric protein, *CPR* C-reactive protein, *CRPM* matrix metalloproteinase-dependent degradation of CRP, *C1M* connective tissue type I collagen turnover

^*^Mean (SD); levels of biomarkers are log_10_-trasformed

Baseline levels of most of the biomarkers were higher in OA cases compared with subjects who remained free of OA during follow-up. At baseline, there were 68 and 185 hip OA and knee OA cases, respectively. At 5-year follow-up, 41 hips (32 new cases) and 111 knees (68 new cases) with incident OA were identified. Overall progression of OA was found in 170 out of 1295 participants who were eligible for OA progression analyses [knee OA cases (n = 122) and/or hip OA cases (n = 53)].

Biomarkers levels were significantly correlated to BMI and age except for uCTX-II with BMI (*p* = 0.37) and CRPM with age (*p* = 0.54) (data not shown). Moreover, there were significant differences in C1M (*p* = 0.003) and uCTX-II levels (*p* <0.0001) between men and women. For age, gender, and BMI-corrected data, it was found that most of the biomarkers at baseline were significantly associated with each other, with the highest correlation between CRP, CRPM, and C1M (Table 2). Urinary CTX-II was not correlated to any of the inflammatory markers.

**Table 2:** Pearson correlations between biomarkers

		CRPM	CRP	COMP	uCTX-II
C1M	Correlation coefficient	0.330	0.580	-0.111	0.033
	*p* value	<0.0001	<0.0001	0.001	0.223
CRPM	Correlation coefficient		0.248	-0.015	-0.004
	*p* value		<0.0001	0.598	0.896
CRP	Correlation coefficient			-0.104	-0.041
	*p* value			0.0001	0.135
COMP	Correlation coefficient				0.159
	*p* value				<0.0001

The correlations were adjusted for sex, age, and body mass index

*CRPM* matrix metalloproteinase-dependent degradation of CRP, *CRP* C-reactive protein, *COMP* cartilage oligomeric protein, *uCTX-II* (urinary) type II collagen degradation, *C1M* connective tissue type I collagen turnover

### Incidence and progression analyses

We observed a significant increased risk for incident OA with higher levels of uCTX-II, COMP, and CRP, which all remained significant in the full model including all markers and covariates (Table 3). Incident OA cases showed a trend toward higher levels of CRPM at baseline, but this did not reach significance level. Overall progression of OA was associated with uCTX-II, COMP, CRP, and CRPM levels. We found that the reported associations were independent of other biomarkers and remained significant in the full models.

**Table 3:** Adjusted odds ratios (OR) and 95 % confidence intervals (CI) from logistic regression models for incident and progression of osteoarthritis (OA) in relation to biomarkers levels

	Incidence of OA, n = 88/955^†^		Progression of OA*, n = 170/1125^‡^	
	OR (95 % CI)	*p* value	OR (95 % CI)	*p* value
uCTX-II	1.3 (1.0–1.7)	0.05	1.3 (1.1–1.5)	0.01
COMP	1.3 (1.1–1.6)	0.02	1.2 (1.04–1.5)	0.02
CRPM	1.2 (1.0–1.5)	0.07	1.3 (1.1–1.5)	0.01
C1M	1.1 (0.9–1.4)	0.23	1.1 (1.0–1.4)	0.10
CRP	1.5 (1.2–1.9)	0.0003	1.3 (1.1–1.5)	0.01
Full model				
uCTX-II	1.3 (1.0–1.7)	0.05	1.3 (1.1–1.5)	0.01
COMP	1.3 (1.0–1.6)	0.03	1.2 (1.02–1.5)	0.03
CRPM	1.2 (0.9–1.5)	0.17	1.2 (1.01–1.5)	0.04
C1M	0.8 (0.6–1.1)	0.11	1.0 (0.8–1.2)	0.67
CRP	1.8 (1.3–2.4)	0.0003	1.3 (1.02–1.6)	0.03

OA incident defined as Kellgren and Lawrence (KL) ≥2 at knee and/or hip; models were adjusted for age, sex, and body mass index; full models include all biomarkers adjusted for age, sex, and body mass index

*uCTX-II* (urinary) type II collagen degradation, *COMP* cartilage oligomeric protein, *CRPM* matrix metalloproteinase-dependent degradation of CRP, *C1M* tissue type I collagen turnover, *CRP* C-reactive protein

^†^Case number/control number; ^‡^progressed/no-progressed; *additionally adjusted for prevalent OA

We stratified the analysis for incidence and progression according to the affected joint (knee and hip OA). However, statistical power was limited per stratum leading to wider confidence limits and nonsignificant results (Additional file 1). Additional joint-based generalized estimating equation analyses between incidence and progression of OA and individual or combined markers showed similar results (data not shown).

Cross-sectional logistic regression analyses adjusted for age, sex, and BMI between baseline OA status and CRPM (OR = 1.0, 95 % CI = 0.8–1.1, *p* = 0.58), CRP (OR = 1.0, 95 % CI = 0.8–1.1, *p* = 0.70), and C1M (OR = 1.0, 95 % CI = 0.9–1.2, *p* = 0.92) levels showed no significant associations. Moreover, we found a positive association, albeit nonsignificant, between hip pain and CRPM levels (OR = 1.6, *p* = 0.14), and between knee pain and CRP (OR = 1.3, *p* = 0.10), and C1M levels (OR = 1.3, *p* = 0.09) adjusted for age, sex, and BMI among OA baseline cases.

**Fig. 1:**
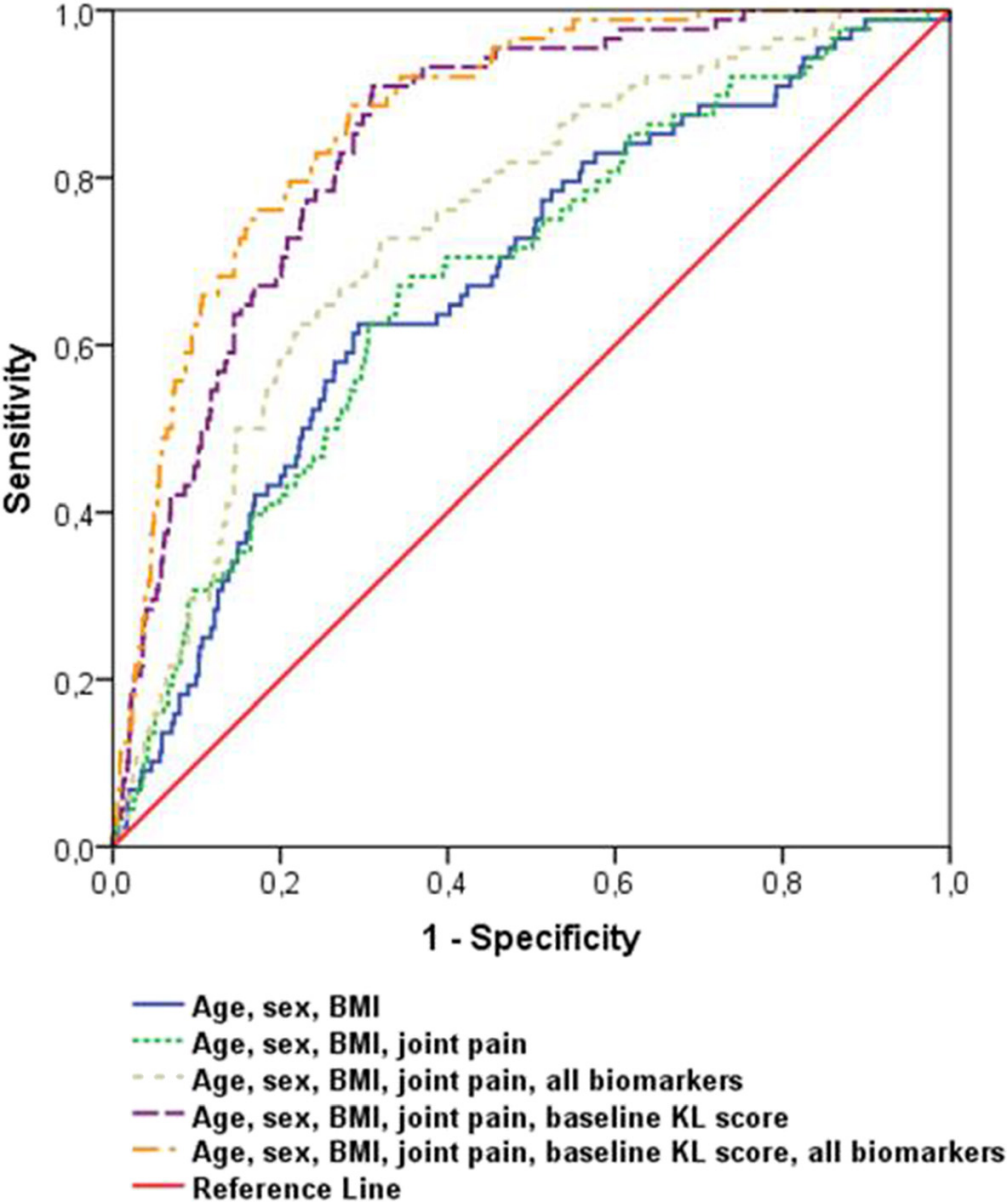
ROC curves of the risk prediction models for incident osteoarthritis

**Fig. 2:**
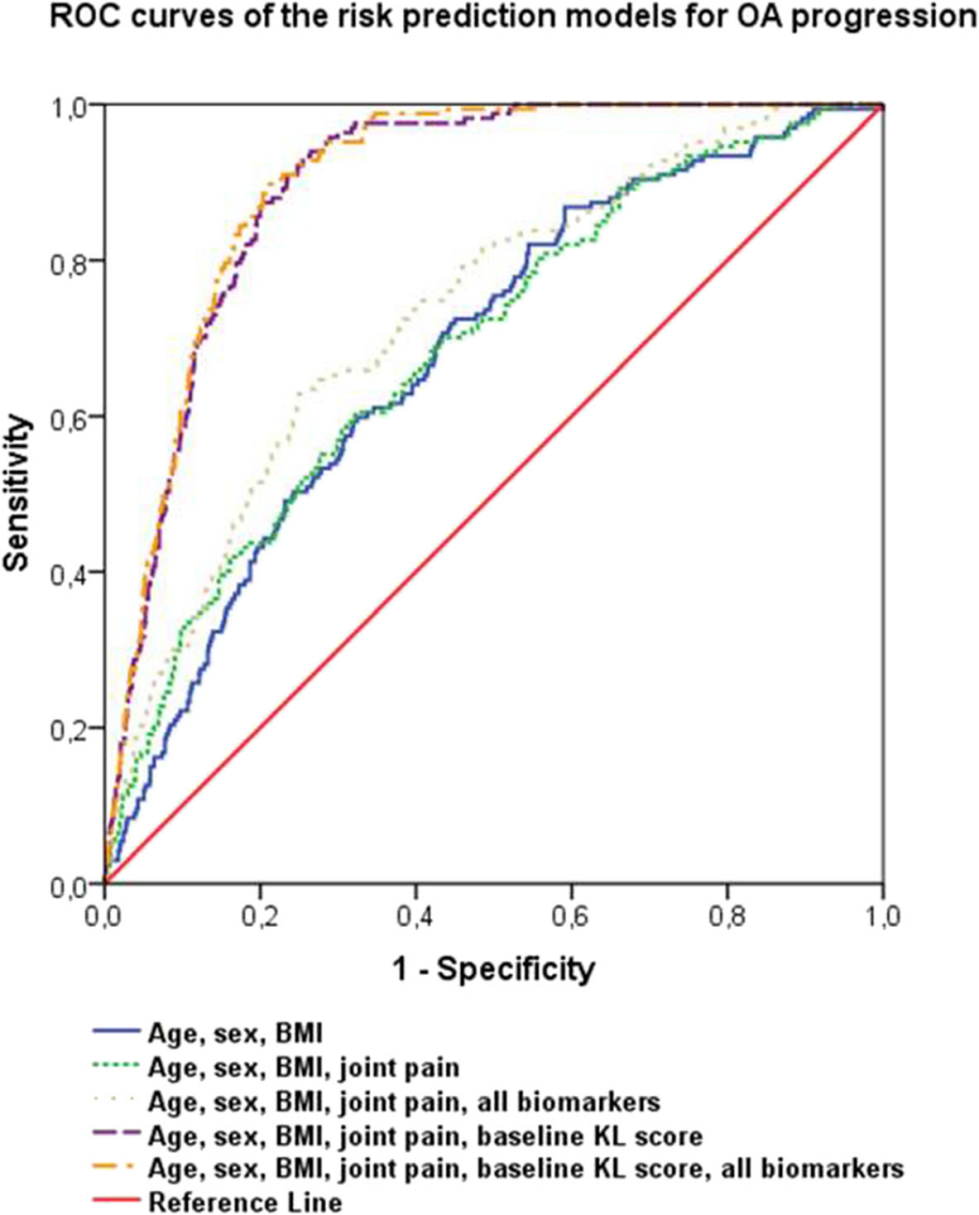
ROC curves of the risk prediction models for osteoarthritis progression

ROC curves of individual markers did not show higher discriminative ability for C1M, CRPM, or CRP models compared with uCTX-II in all OA analyses except a slightly higher prediction ability of CRP for total OA and hip OA incidence. ROC curves of the models including demographic variables resulted in an AUC of 0.68 for OA incidence (Fig. 1 and Additional file 2) and progression (Fig. 2 and Additional file 2). Adding the joint pain variable to the models did not improve it, while subsequent addition of all biomarkers added considerable predictive value for OA. Doubtful baseline KL score of one is the best predictor of future OA, even better than age, gender and BMI alone.

## Discussion

We here evaluated two novel biomarkers for tissue inflammation (C1M and CRPM) for their ability to predict incidence and progression of OA after 5 years of follow-up in a population-based setting. We show that CRPM is associated with incidence and progression of OA. Moreover, CRPM had prognostic value for progression of OA independent from uCTX-II and COMP. In the full statistical model, CRPM and CRP proved to have similar prognostic value as uCTX-II and COMP. Together the biomarkers added significant prognostic power to the known risk factors of age, gender and BMI.

To date, several studies have reported that levels of uCTX-II and COMP are associated with OA and progression of OA, suggesting them as the most promising OA biomarkers [5, 7–10, 28–31]. Consistently, we found that uCTX-II and COMP levels were correlated with increased risk and progression of OA, confirming our previous finding among participants of RS-I [7, 10]. However, despite substantial clinical investigations, none of these markers have yet been proven to be of definite clinical value [32]. Different outcome measures and study designs as well as limited sample size of these studies have resulted in inadequate discriminating ability to differentiate between individual patients and controls (diagnosis) or between patients with different disease severities (burden of disease), and to predict prognosis in individuals with or without osteoarthritis (prognosis) [33].

Our study showed that CRP levels were related to the risk and progression of OA. In our previous study with RS-I participants, an association between CRP levels and incidence of hip OA was seen as well [12]. Data on hip OA progression was limited in that review. A recent review and meta-analysis of 32 studies showed no significant associations between CRP levels and progression of OA [defined as either exacerbation of joint space narrowing (JSN) or TJR], while CRP levels were significantly associated with pain and decreased physical function. Therefore, it was suggested that CRP may be elevated in OA patients, but probably plays a greater role in symptoms rather than radiographic changes in OA [14]. In our study, we observed no significant associations between knee and hip pain and CRP levels among prevalent cases at baseline when controlled for BMI. It should be noted that there was significant heterogeneity due to the quality and methodology of the studies included in the meta-analyses and the result should be regarded with caution.

It has been shown that the CRPM level is elevated in OA patients compared to the healthy adult reference range, and that the inflammatory burden is independent of disease severity (KL score) [15]. In our study, we observed progression of OA to be associated with higher levels of CRPM. Moreover, the association seemed to be independent of CRP. Previous studies did not support the role of systemic inflammation in OA etiology and progression [14], however, CRPM, a degradation product of CRP, and a possible biomarker of chronic tissue inflammation, might point toward a local low-grade inflammation to play a role in a subset of individuals with OA. Our results suggest that this subset is more prone to radiographic OA progression.

We observed no association between levels of C1M and incidence and progression of OA in both biomarker-specific and full models. The collagen biomarker C1M was found to be higher in OA patients with an elevated inflammatory burden as measured by high-sensitivity CRP [15] and CRPM [16]. Previous analysis of C1M in RA has shown that the biomarkers were associated with structural progression measured by JSN [15]. Moreover, a recent ex vivo experiment showed that under pro-inflammatory conditions serological biomarkers C1M, MMP-mediated degradation of collagen type III (C3M), and active MMP-3 may originate from the inflamed synovial membrane (Kjelgaard-Petersen CF, Bay-Jansen AC, Christiansen T, Ladel C, Karsdal MA, Siebuhr AS. Novel synovitis biomarkers: TNF-α and IL-1β include MMP-mediated degradation of collagen type I and III and active MMP-3 and -9 in synovial membrane explants. 2014, Submitted). Our study showed significant positive linear association between C1M levels and CRPM, and CRP levels adjusted for age, sex, and BMI among baseline OA cases (data not shown). These findings illustrates that connective tissue turnover in OA (i.e., C1M levels) is increased with inflammation and, together with our findings of CRP and CRPM, provide more support to the importance of local inflammation in OA pathogenesis in a subgroup of patients.

Identification of subjects at a high risk of OA is necessary for preventive strategies. Biochemical markers might help in identification of subgroups of OA patients with higher risk of progression. Age, gender, and BMI are already rather strong predictors of OA risk and progression at older age in the population studied and in other investigations [10]. In a previous study among participants of RS-I, a prognostic model for incident knee OA was developed based on clinical, genetic and biochemical (uCTX-II) risk factors [34]. The study showed that these risk factors combined had a relatively low predictive value for knee OA. In contrast, a model including doubtful minor radiographic degenerative features (KL score = 1) reached a good predictive value. Consistently, in our study, baseline KL score of one was the best predictor of future OA, and even better than age, gender and BMI alone. Addition of well-established biochemical markers together with tissue inflammation biomarkers added moderate predictive value to most of our models. Moreover, for the first time, we showed that a marker of MMP-dependent inflammation is able to predict progression of OA independent of established biomarkers uCTX-II and COMP. Future investigations are needed to identify the potential usefulness of the tissue inflammatory biomarkers in OA prediction in a clinical setting.

Radiographic OA bears little relationship to the illness characterized by joint pain and functional impairment [35, 36]. Different factors (i.e., bone marrow lesions, joint effusion, psychological factors, comorbidities) and mechanisms of joint pain (i.e., nociceptive pain, neuropathic pain, and central pain sensitization) have been described in OA patients [37, 38]. A recent study among OA patients showed the correlations between central pain mechanisms (temporal summation and pain modulation) and CRPM, independently of age, gender, BMI, and hsCRP [37]. Consistently, we found a positive association, albeit nonsignificant, between hip pain and CRPM levels, and between knee pain and CRP and C1M levels. These trends support the role of inflammation as a possible underlying mechanism of joint pain in OA, but more power is needed to definitely prove this suggestion.

There were considerable correlations between different markers reflecting turnover of cartilage (CTX-II, COMP, and C1M) and inflammatory markers (CRP and CRPM). Therefore, simultaneous modeling of several markers could provide more insight into the role of individual markers in OA progression. Moreover, due to the longitudinal design of the study, we were able to assess the potential predictive value of the markers to predict disease risk and progression. Controlling for confounder factors including age, sex, BMI, smoking status, education, and diabetes is another strength of our study. Our study, however, had some limitations that must be taken into account. First of all, biomarkers were only assessed in baseline samples. Serial biomarker assessments could also be very informative on the natural dynamics of each biochemical marker and on disease status. Moreover, the biomarker contributions from OA need to be distinguished from the contributions of normal and age-related bone and cartilage turnover, and other conditions affecting the biomarkers levels; this is currently a primary limitation for all systemic (serum and urine) biomarker measures [33]. However, individuals with rheumatoid arthritis or other inflammatory arthropathies were excluded from our analyses. Additionally, we adjusted the analyses for BMI and age, two major factors which affect the biomarkers levels. Second, OA definition was based on KL grades, which conflate osteophytes, and JSN and some studies have shown that biomarker associations can be different with respect to these related but different features [39]. Third, knee OA was defined using anteroposterior radiographs of the knee. Therefore, patellofemoral joint OA was not taken into account in the study. Moreover, uncontrolled occurrence of misalignment of the medial tibial plateau and central X-ray beam in standing anteroposterior radiographs of the knee might result in an underestimation of the rate and homogeneity of JSN in knee OA [40]. Fourth, pain was assessed by questionnaire and not by more precise methods such as Western Ontario and McMaster Universities Arthritis Index pain score or visual analog scale. Fifth, controls were free of OA at the joint site studied but were allowed to have OA at other joint sites. For example, if knee OA was studied, controls had to be free of knee OA but were allowed to have hip OA. In addition, the inference of the relevant relationships between serum or urine biomarker concentrations and disease of specific joints is complicated by the fact that our OA patients might have disease in other joints such as hands or spine joints. Sixth, several studies have suggested that risk factors for incidence of OA may be different from risk factors for progression [41]. This could be due to heterogeneity in OA structural pathology (e.g., bony proliferation versus cartilage loss), limitations of imaging, which may result in different sensitivities to the structural features, limited sample size of the studies that examined the risk factors, and that the risk factors may affect disease differently at different disease stages [41]. Although we had a reasonable sample size for total OA incidence and progression analyses, the statistical power was limited to identify which factors operate at different disease stages and for analyses of OA phenotypes leading to wider confidence limits. Finally, although we covered several confounding variables, we could not exclude bias due to unmeasured confounding factors as well as health-based selection bias as we used a subset of RS-II participants who had follow-up data. These subjects were probably more mobile to visit the center and survived in the follow-up period.

## Conclusions

Our study confirmed that the uCTX-II and COMP concentrations are associated with incidence and progression of radiographic OA. Moreover, we showed for the first time that a MMP-dependent tissue-inflammation marker predicts the risk of OA progression independent of established biomarkers uCTX-II and COMP at the 5-year follow-up. This indicates that inflammation is associated with disease progression in OA and that inflammation has pathological relevance in OA. Further prospective studies are needed to confirm this association for OA.

## Additional files

Additional file 1: Table S1. Adjusted odds ratios (OR) and 95 % confidence intervals (CI) from logistic regression models for incident and progression of knee and hip OA in relation to biomarkers levels. (PDF 467 kb) Additional file 2: Table S2. Discrimination, described as AUC (95 % confidence interval), for the risk prediction models of osteoarthritis (OA). (PDF 59.8 kb)

## Abbreviations

AS: ankylosing spondylitis
AUC: area under the curve
BMI: body mass index
C1M: connective tissue type I collagen turnover
CI: confidence intervals
COMP: cartilage oligomeric protein
CRP: C-reactive protein
CRPM: matrix metalloproteinase-dependent degradation of CRP
GEE: generalized estimating equation
hs: high-sensitivity
JSN: joint space narrowing
KL: Kellgren and Lawrence
MMP: matrix metalloproteinase
OA: osteoarthritis
OR: odds ratios
RA: rheumatoid arthritis
RS: Rotterdam Study
ROC: receiver operating characteristic
TJR: total joint replacement
uCTX-II: (urinary) type II collagen degradation
