# Supplementary material for: Association between biomarkers of tissue inflammation and progression of osteoarthritis: evidence from the Rotterdam study cohort
Source: Arthritis Res Ther. 2016 Apr 1;18:81. doi: 10.1186/s13075-016-0976-3 (PMC4818486; doi:10.1186/s13075-016-0976-3)
Supplement: Additional file 1: Table S1. — Adjusted odds ratios (OR) and 95 % confidence intervals (CI) from logistic regression models for incident and progression of knee and hip OA in relation to biomarkers levels. (PDF 467 kb) [file 13075_2016_976_MOESM1_ESM.pdf]

Supplementary Table 1. Adjusted odds ratios (OR) and 95% confidence intervals (CI) from logistic regression models for incident and progression of knee and hip OA in relation to biomarkers levels

	**Knee OA**				**Hip OA**			
	**Incidence**, n=68/1029†		**Progression*,** n=122/1157‡		**Incidence**, n=32/1191†		**Progression*,** n=53/1233‡	
	**OR (95% CI)**	***p*-value**	**OR (95% CI)**	***p*-value**	**OR (95% CI)**	***p*-value**	**OR (95% CI)**	***p*-value**
**uCTX-II**	1.2 (0.9-1.6)	0.13	1.2 (1.01-1.5)	0.04	1.4 (1-2.1)	0.05	1.4 (1.1-1.9)	0.02
**COMP**	1.6 (1.2-2.04)	0.0004	1.4 (1.2-1.7)	0.001	0.8 (0.6-1.1)	0.23	0.91 (0.7-1.2)	0.54
**CRPM**	1.2 (0.9-1.5)	0.21	1.2 (1.01-1.5)	0.05	1.3 (0.9-1.8)	0.17	1.2 (0.91-1.7)	0.19
**C1M**	1.1 (0.9-1.4)	0.47	1.1 (0.93-1.4)	0.23	1.2 (0.9-1.6)	0.32	1.1 (0.87-1.5)	0.35
**CRP**	1.4 (1.1-1.8)	0.01	1.2 (1.01-1.5)	0.04	1.5 (1.1-2.2)	0.02	1.3 (0.94-1.7)	0.12
**Full model**								
uCTX-II	1.2 (0.9-1.6)	0.19	1.2 (0.97-1.5)	0.09	1.5 (1.1-2.2)	0.02	1.5 (1.1-2.04)	0.009
COMP	1.6 (1.2-2.04)	0.001	1.4 (1.14-1.7)	0.002	0.8 (0.6-1.1)	0.18	0.9 (0.66-1.2)	0.40
CRPM	1.1 (0.9-1.5)	0.37	1.2 (0.95-1.5)	0.13	1.2 (0.8-1.8)	0.28	1.2 (0.85-1.6)	0.32
C1M	0.8 (0.6-1.1)	0.25	0.96 (0.8-1.2)	0.77	0.8 (0.5-1.2)	0.25	0.94 (0.7-1.3)	0.74
CRP	1.7 (1.2-2.3)	0.004	1.3 (0.98-1.7)	0.07	1.7 (1.1-2.8)	0.03	1.3 (0.89-1.9)	0.18

All models were adjusted for age, sex, body mass index, and follow-up time; Full models include all biomarkers adjusted for age, sex, body mass index, and follow-up time; †case number/ control number; ‡progressed/no-progressed; *additionally adjusted for prevalent OA; OA incidence defined as KL≥2 at left and/or right joint; subjects were free of hip and knee OA at baseline for hip and knee incidence analyses, respectively.
