# Supplementary material for: Association between biomarkers of tissue inflammation and progression of osteoarthritis: evidence from the Rotterdam study cohort
Source: Arthritis Res Ther. 2016 Apr 1;18:81. doi: 10.1186/s13075-016-0976-3 (PMC4818486; doi:10.1186/s13075-016-0976-3)
Supplement: Additional file 2: Table S2. — Discrimination, described as AUC (95 % confidence interval), for the risk prediction models of osteoarthritis (OA). (PDF 59.8 kb) [file 13075_2016_976_MOESM2_ESM.pdf]

Supplementary Table 2. Discrimination, described as AUC (95% Confidence Interval), for the risk prediction models of osteoarthritis (OA)

	**Incident OA**	**Progression of OA**
Age, sex, BMI	0.677 (0.620-0.735)	0.681 (0.639-0.722)
Age, sex, BMI, joint pain	0.682 (0.624-0.740)	0.688 (0.646-0.731)
Age, sex, BMI, joint pain, all biomarkers	0.746 (0.695-0.796)	0.731 (0.691-0.771)
Age, sex, BMI, joint pain, baseline KL score	0.845 (0.809-0.881)	0.893 (0.874-0.913)
Age, sex, BMI, joint pain, baseline KL score, all biomarkers	0.872 (0.839-0.905)	0.899 (0.880-0.918)

body mass index (BMI); Kellgren-Lawrence (KL); (urinary) type II collagen degradation (uCTX-II); cartilage oligomeric protein (COMP)
